# A Mixed-Methods Analysis of Motivational Dynamics and Strava Use in Active Club Runners

**DOI:** 10.3390/bs16020224

**Published:** 2026-02-03

**Authors:** Malene Rob Kolnes, Karsten Øvretveit

**Affiliations:** 1 Department of Education and Sports Science, Faculty of Arts and Education, University of Stavanger, 4036 Stavanger, Norway; mr.kolnes@stud.uis.no; 2 Department of Public Health and Nursing, Faculty of Medicine and Health Sciences, Norwegian University of Science and Technology, 7491 Trondheim, Norway

**Keywords:** achievement goals, self-efficacy, Strava, running, motivation

## Abstract

The application Strava is widely used among runners, yet its influence on motivational processes remains unclear. This study examined endurance sport self-efficacy, achievement goals, fitness indicators, and Strava use in 225 active club runners using validated quantitative instruments and qualitative survey data. Self-efficacy and achievement goal scores were generally high. Greater endurance capacity was associated with higher self-efficacy and task-approach goals. Strava settings and subscription status were not associated with motivational outcomes; however, runners who had deleted training sessions due to perceived slow running pace scored higher on other-avoidance goals. Qualitative findings showed that Strava can enhance training through feedback, routine building, and social connection, while also introducing pressure, comparison, and stress, particularly during injury or reduced performance. Several participants reported adapting their use of the app to preserve motivation. Overall, Strava’s motivational impact appears context dependent and shaped by both its features and individual usage patterns.

## 1. Introduction

In recent years, the sport of running has experienced a boom in popularity. In certain parts of the world, it is one of the most popular sports to engage in (Hulteen et al., 2017; Janssen et al., 2017), and participation in races such as the half and full marathon is also increasing (Knechtle et al., 2016; Wilke et al., 2019). Alongside the growing popularity of running, the use of training technology and software in leisure activities is also becoming more common (Kuru, 2016; Whelan & Clohessy, 2021). The use of technology, such as software applications, to track training sessions may be motivated by improving performance or maintaining adherence (Karahanoğlu et al., 2021), but the social feature in such applications is associated with how users engage in physical activity (Petersen et al., 2020).

The online software service, Strava, provides one of the most widely used fitness applications for activity tracking, with over 150 million users in over 185 countries (Strava, 2025). In addition to exercise metrics, it has social networking features that may affect training patterns in activities such as running (Franken et al., 2023). Strava can also add to the social connection that can occur in run groups and clubs by providing digital connection, communication, and performance comparisons (Tulle et al., 2018). The social aspect can lead to both adaptive and maladaptive behaviors, such as runners curating their own image by not posting slow runs (Russell et al., 2023), or even having others performing activities for you, also known as “Strava Jockeys” (Davidson, 2024).

Several psychological frameworks have previously been used to explore engagement and motivation dynamics in physical activity. Self-efficacy (Bandura, 1977) has been shown to predict physical performance (Horcajo et al., 2022), and it has been linked to physiological improvements, such as in maximal oxygen uptake ($\dot{V}$O_2max_) (Montes et al., 2018), which is a key determinant of endurance performance. Furthermore, the way athletes perceive physical competence can influence both performance and adherence to sports. Previous research has shown that runners tend to adopt intrapersonal achievement goals (Delrue et al., 2016; Xiang et al., 2004), which is consistent with adaptive behavior patterns.

Due to their relative novelty and increasing use in recreational sport, research on the impact of software applications on sport behavior is warranted. Accordingly, the aim of the present study was to characterize self-efficacy and achievement goals in active runners and to investigate associations between these constructs and the use of Strava as a training tool and social network, including potential sex differences and the influence of performance indicators and training experience.

## 2. Materials and Methods

### 2.1. Participants

Active, regional run clubs were identified through the website of the Norwegian Olympic and Paralympic Committee and Confederation of Sports (NIF). All eligible clubs were contacted and invited to participate in the study. Following the recruitment of active runners in registered regional clubs, we invited other active runners in the region belonging to clubs that originally did not appear on the NIF website. In total, 225 active club runners (43% women) aged 18 to 79 years (mean 40.5 ± 10.3 years) participated in this study (Table 1). All participants provided written informed consent. Data collection procedures were reviewed by and carried out in accordance with the Norwegian Agency for Shared Services in Education and Research.

### 2.2. Measurement Procedures

Data collections were handled by questionnaires created with nettskjema.no, a survey solution developed and hosted by the University of Oslo (nettskjema@usit.uio.no). To be able to quantify exercise behavior and performance, as well as further explore underlying motivations, we analyzed both quantitative and qualitative data. We developed a comprehensive questionnaire that was distributed via a secure, online platform. At the end of the quantitative questionnaire, the participants were encouraged to respond to an open-ended question. These qualitative data were analyzed in accordance with Braun and Clarke (2022).

In addition to items addressing training habits and other metrics, such as estimated $\dot{V}$O_2max_, two validated instruments were translated and included in the questionnaire. The Endurance Sport Self-Efficacy Scale (ESSES) is an 11-item, non-hierarchical, unidimensional scale that was developed and validated to assess self-efficacy beliefs in relation to endurance sport performance (Anstiss et al., 2018). The 3 × 2 Achievement Goal Questionnaire for Sports (3 × 2 AGQ-S) (Mascret et al., 2015) defines and measures six distinct achievement goals in sport settings and aims to capture athletes’ approach and avoidance motives across task-based, self-based, and other-based definitions of competence.

Prior to the main data collection, a pilot study (n = 5) was conducted for a preliminary, independent assessment of the questionnaire. The participants were encouraged to provide feedback on items that appeared unclear or incomplete, as well as the perceived duration and workload of completing the form. The main study data were collected over six weeks, from 1 October 2024 through 15 November 2024.

### 2.3. Statistical Analysis

Data normality was assessed with the Kolmogorov–Smirnov test and visual inspection of histograms. Because normality assumptions were violated, groups were compared using the Mann–Whitney U and Kruskal–Wallis tests. Associations between variables were determined using Spearman’s rank correlation coefficients (r_s_). In analyses with physical fitness, the athletes were stratified by their watch-estimated $\dot{V}$O_2max_ (_WE_$\dot{V}$O_2max_) based on established normative values (The Cooper Institute, 2017). The factor structure of the translated versions of ESSES and 3 × 2 AGQ-S was investigated using exploratory factor analysis (EFA). We used the commonly applied rotation method, Promax, to interpret and identify the underlying structure (Dien, 2010; Grieder & Steiner, 2022), with both scales showing acceptable psychometric properties in the present sample. Reliability was assessed using Cronbach’s alpha (α). A *p*-value < 0.05 was considered statistically significant. All statistical analyses were performed with IBM SPSS Statistics v. 29 (IBM Corp., Armonk, NY, USA). Figures were made with GraphPad Prism v. 10 (GraphPad Software, San Diego, CA, USA).

## 3. Results

### 3.1. Quantitative Findings

Several sex differences were observed among the participating runners, including in _WE_$\dot{V}$O_2max_, age, and the duration of Strava use (Table 1). On average, women also scored lower on ESSES (72.7 ± 13.8 vs. 77.6 ± 11.2; *p* = 0.014) and task-approach goals (4.6 ± 1.0 vs. 4.9 ± 1.2; *p* = 0.020) compared to men. We found several associations between ESSES and achievement goals, as well as Strava use metrics (Table 2). ESSES scores were generally high in the sample, with no difference across age groups.

**Figure 1:**
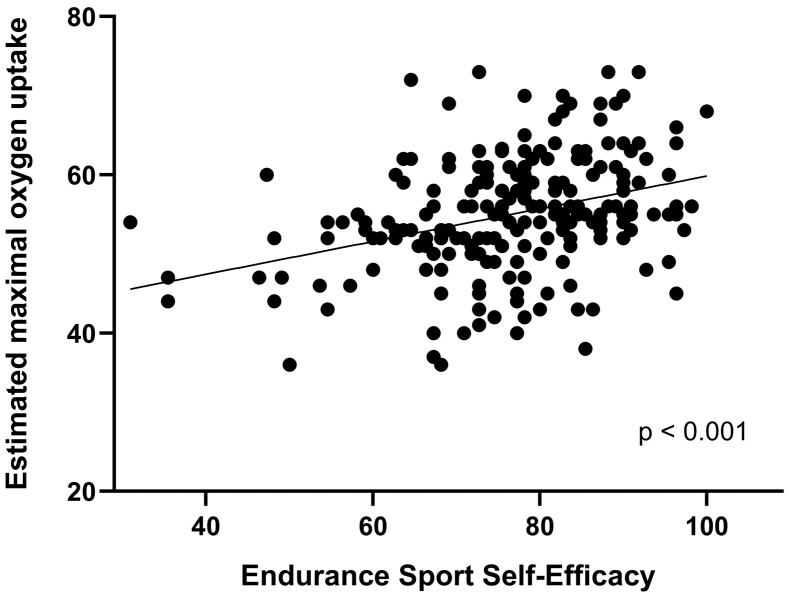
Scatterplot showing the association between endurance sport self-efficacy (ESSES) and watch-estimated maximal oxygen uptake.

Over 98% of the participants reported training with a sports watch, out of which 87% used a Garmin. Generally, higher _WE_$\dot{V}$O_2max_ was associated with higher ESSES scores (Figure 1), with runners in the highest _WE_$\dot{V}$O_2max_ groups (3–5) scoring higher on ESSES compared to lower groups (*p* < 0.05). A similar pattern was also found for task-approach goals (Table 2).

To further explore the association between actual performance level and motivational outcomes, we tested correlations between personal best running times and these outcomes. An inverse correlation was observed between ESSES and personal bests on the 3000 m (r_s_ = −0.449), the 5000 m (r_s_ = −0.404), the 10,000 m (r_s_ = −0.388), and the half marathon (r_s_ = −0.342; all *p* < 0.01), but not the full marathon (*p* > 0.05). Similar relationships were observed between all reported personal bests and task-approach goals (r_s_ = −0.385 to −0.429; *p* < 0.01).

ESSES also correlated positively with the number of years on Strava and the number of Strava followers and people that the runner followed, which can be considered proxies for the use of Strava’s networking features (Table 2). Other Strava use metrics, such as having a paid subscription, a closed profile or reporting concerns about how running tempo would show up on Strava during a training session were not associated with either ESSES or achievement goal orientations. However, runners who reported having deleted a training session due to running pace (3.62 ± 1.61) scored higher on other-avoidance goals compared to those who had never deleted a session (2.65 ± 1.35; *p* < 0.05).

### 3.2. Qualitative Findings

Analyses of the qualitative data resulted in four overarching themes: Strava as a training tool and source of motivation, the social aspect of Strava, its challenges and negative impact, and the customization and individualization of use.

#### 3.2.1. Strava as a Training Tool and Source of Motivation

Participants reported that Strava played an important role as a practical training tool and a source of motivation for many runners. Tracking was highlighted as a central function of the application, where the automatic recording of distance, pace, and heart rate provided a clear overview of training sessions and progress. One respondent described it as follows:

“*[Strava is a] good training tool. It provides an excellent overview of my runs. Useful for comparing my own sessions with similar sessions done previously. Perfect for, for example, test runs or other hard workouts where I repeat the same session or route again and again.*”

(Male, 28)

Other respondents added:

“*I like having a log of the past so that I can see my development. Strava is a wonderful tool for logging and tracking progress.*”

(Male, 40)

“*Strava is motivating in terms of logging training and reviewing what I have done in similar sessions previously.*”

(Male, 39)

“*It is one of the most important elements for me (as a recreational runner) to maintain continuity in my training.*”

(Male, 38)

“*It is useful to see how my condition changes from day to day and how my body responds to different types of training.*”

(Male, 41)

“*For those of us who run short distances and only on the track, it is a simple tool for logging training, nothing more.*”

(Male, 65)

A common theme among several respondents was the use of Strava for logging and monitoring personal progress, which was viewed as a motivation for maintaining routines and continuity. Some respondents also noted that their Strava use was limited solely to logging, without additional functional or social significance.

#### 3.2.2. The Social Aspect of Strava

Several respondents reported the social aspect of Strava to be an important source of motivation, such as through receiving kudos and comments. One respondent explained:

“*Strava is motivating for my own training because of the kudos and comments.*”

(Male, 48)

Another respondent emphasized how Strava differed from other training platforms by facilitating interactions between both friends and club members:

“*From my own experience, Strava—compared to Garmin Connect, for example—is more engaging precisely because it makes it easier to connect with both close friends and club members. I notice that it gives me a boost, even though I wish I could say it doesn’t matter. But it drives me in a positive way in terms of training, even if it is not necessarily to perform for anyone other than myself.*”

(Male, 30)

Additionally, several respondents found it inspiring to follow the training of others, such as observing training habits, routes, and progression. This was by many considered a source of both inspiration and learning:

“*You can see other people’s training and draw both inspiration and lessons from it. The function for finding routes on the map is also brilliant. It is probably motivating to many. The little extra push needed to get out the door to train.*”

(Male, 28)

“*Ambitions are no longer that important at my age, rather it is about avoiding injuries, maintaining an acceptable physical activity level, and keep up with and feel connected to current and former sports friends, preferably through Strava instead of Facebook.*”

(Male, 79)

“*I prefer Garmin Connect. It’s free and provides more detailed data. I mostly use Strava to give kudos to friends, and to see if they are training. It gives us something to talk about when we train together.*”

(Male, 47)

“*Strava, when used correctly, is a useful tool for finding routes and even running partners, as well as for communication within clubs and among acquaintances or training partners.*”

(Female, 44)

Although several respondents reported that Strava served as a positive source of motivation, some also pointed out that its social media features could have negative effects. One respondent noted:

“*I have had Strava for several years, but I did not start using it actively until I began running regularly and with clear goals. I have noticed that the app has taken more of my attention over time, and I have considered not using it for around a year. It is nice to receive kudos and followers, but lately I have reached a point where it can sometimes feel a bit exhausting to get kudos on my own sessions and the thought of people watching, and consequently to feel that I should give kudos and follow others’ sessions to ‘give something back.’ At the same time, it is valuable to have a platform where one can share results and training sessions and draw inspiration from like-minded individuals. I do not think it is negative that Strava can provide some external motivation. I think Strava is positive, while it is important to reflect on how it influences our thought patterns and use of time.*”

(Male, 31)

Other respondents pointed specifically to challenges associated with Strava’s social features and how these could have negative consequences for users:

“*It’s not healthy to have notifications turned on! One should avoid this and the chase for likes. Otherwise, it’s an excellent tool!*”

(Female, 35)

“*Lately it feels as though Strava has become more of a social platform. This can lead people to trust others’ performances less, as some may embellish their efforts or cheat, for example with Strava jockeys. In the past, Strava felt genuine and reliable, but perhaps the future will unfortunately not be like this?*”

(Male, 45)

“*Strava encourages improper training. Everything becomes a competition, every day, even during training.*”

(Male, 73)

“*Gotten tired of sharing things! If it motivates some people, great. But over time it tends to become stressful, and one becomes more concerned with ‘how it looks’ than simply training.*”

(Male, 52)

Several respondents described Strava as a social and motivating training tool that facilitated contact with both friends and the athletic community. Interacting through kudos and comments could lead to motivation, inspiration, and a sense of belonging. Some respondents also noted that this form of external motivation and the need for social validation could interfere with their initial enjoyment of training. There were also critical reflections regarding how the application might influence individuals’ thought patterns and distort reality.

#### 3.2.3. Challenges and Potential Negative Impact

Despite many positive aspects of Strava being highlighted, several respondents also pointed to its challenges and potential negative impact. A central concern was the feeling of pressure and comparison with others. This led some individuals to avoid using the application for fear of becoming overly preoccupied with their own or others’ training. Some respondents also expressed concern that the application might shift the focus from personal development and health toward seeking recognition from others:

“*I think many people run more for Strava (numbers, kudos, nice statistics)/for others (to show what they can do) than for themselves. Being able to run far, fast, and often may perhaps be considered as a form of ‘status’ rather than something enjoyable and health-promoting.*”

(Male, n/a)

“*It can easily become addictive. For me, in a positive way, but I get that others may quickly start comparing themselves with others, and then Strava can work against its intended purpose.*”

(Male, 38)

Some participants also reported that Strava contributed to performance pressure, particularly in vulnerable situations, such as during injury:

“*I am currently dealing with long-term injury problems and have therefore logged off Strava to avoid negative feelings related to my lack of effort. I notice myself reflecting on whether I should stop using Strava altogether, even though it has been a fantastic social support network, I now experience more performance-related stress than before. I suspect that influencers have spoiled some of the fun with their polished images, Strava was better before ‘everyone’ joined!*”

(Female, 43)

Others pointed out that if you experience pressure from Strava, it is often individual perceptions that underlie this experience rather than the application itself:

“*If someone experiences it as pressure, they should look inward. Strava is not the cause of that person’s challenges. Personally, I am not very concerned with social media and therefore have no need to be on Strava in addition to the social media platforms we were already pushed into using from the beginning.*”

(Female, 44)

Overall, the participants indicated that Strava was not necessarily perceived as positive by everyone, and that the main source of negative influence stemmed from social exposure and comparison with others.

#### 3.2.4. Customization and Individualization of Strava Use

Several respondents expressed the need for individual adaptation in how they used Strava. Some viewed Strava as a useful tool for documenting and analyzing their own training, while others adjusted their use to avoid negative effects. For some individuals, their use of Strava had changed over time. One respondent described a shift in which Strava had previously contributed to stress, but was later approached in a more personalized and constructive way:

“*In the past, I was stressed by Strava and by the running speed of others. Now I am able to train in a way that is right for me and no longer compare myself to others.*”

(Female, 46)

Others opted to reduce their engagement by limiting the visibility of their sessions and minimizing their interaction with other users. For many, adapting their Strava use involved determining how the application could be used in a way that yielded a positive benefit.

“*As long as using Strava gives me something, I will continue to use it, but I put it away if it becomes a burden.*”

(Male, 43)

“*It must be used correctly to be motivating, while at the same time not becoming something one gets too fixated on and the numbers that come with it.*”

(Male, 27)

“*I have not shared any sessions publicly for the past three months. A relief. Gone into ghost mode. No longer pay attention to others. Saves time and I use the app minimally.*”

(Male, 41)

“*Training is personal and only for me and my coach- for me. Strava, when used correctly, is a useful tool for finding routes and perhaps running partners, as well as within clubs and among acquaintances or training partners.*”

(Female, 44)

“*I use it mostly to see what I have done and participated in. I post photos and videos.*”

(Male, 61)

“*Strava can affect your mindset a bit too much, so I mainly use it for my own monitoring.*”

(Male, 28)

The responses showed that many adapted their Strava use in accordance with their own needs and purposes. While some used the application actively to document training and experience a sense of community, others chose to limit the sharing of sessions in order to avoid comparison and pressure. Several reported to have developed a more deliberate approach to Strava over time, which could be beneficial for achieving individual goals and needs, and avoid it becoming a detriment to training adherence and progress.

## 4. Discussion

By analyzing quantitative and qualitative data, the present study examined motivational dynamics and Strava use among active runners. Overall, endurance sport self-efficacy was generally high, with increasing fitness levels being associated with higher self-efficacy and task-approach scores. Although estimations of fitness levels, such as $\dot{V}$O_2max_ according to a sports watch, should be interpreted with caution, we confirmed these relationships using the participants’ actual running times. Strava was a popular training tool among the participants, with an average use time of over an hour per week. Although many emphasized the beneficial aspects of Strava, some also pointed out potentially problematic aspects, including its social functions.

Men reported higher self-efficacy than women. This is consistent with Wiley et al. (2024), who observed higher self-efficacy among individuals with masculine characteristics. This may partly reflect gender stereotypes related to physical competence and performance (Hively & El-Alayli, 2014). Limited exposure to female role models may also reduce women’s vicarious experiences, which in turn can affect their self-efficacy (Bandura, 1977).

The finding that self-efficacy and fitness metrics, in the present paper represented by higher ESSES scores among those with higher _WE_$\dot{V}$O_2max_, is consistent with previous research (Anstiss et al., 2018; Montes et al., 2018; Øvretveit, 2020). A possible interpretation could be that individuals with stronger beliefs in their own abilities may be more likely to spend more time improving their physical abilities. Conversely, high physical competence may in turn improve self-efficacy through positive experiences caused by the competence levels. Indeed, personal mastery experiences can be an important source of influence on self-efficacy (Bandura, 1977). Although there is usually a divergence between laboratory measurements and estimated measures of fitness, Garmin watches, which most of our sample used, have been shown to have acceptable validity for $\dot{V}$O_2max_ (Carrier et al., 2025; Engel et al., 2025).

The participants generally reported achievement goal scores across all age groups, with a slight signal of a higher score among the oldest participants. This pattern may indicate that experience and longer adherence to training tend to co-occur with adaptive behaviors. The opposite may also be true, that older athletes are part of a selected group that has certain attributes that increase adherence, e.g., a focus on mastery. We also found some differences in achievement goals between running clubs, but as we did not investigate their motivational climate specifically, we did not thoroughly assess club differences. A comparative analysis between running clubs that include measures of their perceived motivational climate, is thus an interesting topic for future research.

The present study shows similar sex-specific achievement patterns as earlier research, such as men scoring higher on task-approach goals (Lochbaum et al., 2020), but differ from Brevig et al. (2024), who reported no meaningful sex differences across any 3 × 2 achievement goal. The level of _WE_$\dot{V}$O_2max_ also appeared to have an association with both ESSES and achievement scores, with the caveat that this metric can also be a proxy for sex differences given the higher values in men. At face value, those in the highest _WE_$\dot{V}$O_2max_ group scored the highest on most achievement goals, including both approach and avoidance goals, which may reflect the competitive context of running where performance is easily quantified and compared. This is consistent with the dual pattern that striving to outperform others can coexist with concerns about maintaining a high performance standard (Elliot & McGregor, 2001; Elliot et al., 2011).

Older runners (70–79 years) also showed relatively high task-approach and other-referenced goals, indicating that personal development and social comparison remain with age. Given the decline in $\dot{V}$O_2max_ with age, a perceived downturn in physical capacity may be accompanied by greater attention to how one’s performance is evaluated by others, although this interpretation is speculative. Finally, differences between clubs suggest a potential divergence in motivational climates. Although perceptions of the motivational climates were not assessed, the clubs that averaged higher task-approach scores may have a more supportive mastery-oriented environment (Ames & Archer, 1988), whereas clubs displaying higher avoidance and self-referenced goals, may cultivate a climate where both improvement and failure-avoidance are prominent motivational drivers.

The qualitative data illustrated how Strava can both support and challenge runners’ motivational dynamics. It was described as a valuable training tool that gave clear performance feedback, comparative data, and input from others. Several participants reported to consciously regulate their Strava use, e.g., hiding sessions or limiting interactions, to preserve motivation and avoid comparison. Social exposure appeared to be particularly stressful in times of injury or reduced performance. Concerns related to authenticity, social norms, and the evolving culture of Strava may point to how digital training environments may be implicated in both adaptive and maladaptive motivational processes.

The qualitative findings provided additional context to the observed quantitative associations. Runners with larger Strava networks reported higher self-efficacy, which the qualitative data suggest may reflect social affirmation and feedback through kudos, comments, and visibility within running communities. Participants described these interactions as motivating and supportive of perceived competence. At the same time, their responses illustrated how the same social exposure could become a source of pressure and evaluative concern, particularly during periods of reduced performance or injury, prompting runners to regulate their engagement through strategies such as hiding activities, limiting interactions, or deleting sessions. This may help explain why social network size was positively associated with self-efficacy at the group level, while avoidance-oriented behaviors were linked to higher other-avoidance goals.

A strength of the present study is the qualitative and quantitative data sources that allowed us to explore motivational dynamics in the context of runners on Strava from different angles. The sample size was also fairly large and comprised active runners from a whole region of Norway, improving the ecological validity of the data. The use of several validated instruments, along with physiological estimates, allowed for direct comparison with past and future research. Several limitations should also be acknowledged. The cross-sectional study design limits our ability to assess the direction of relationships, e.g., if Strava is a cause or effect of certain behaviors. The imbalance between the number of participating men and women may affect the generalizability of the sex-specific findings. Strava use was operationalized using general indicators of exposure and duration, without a detailed assessment of feature-specific or motivation-driven engagement, which constrained more detailed analyses of usage patterns. The qualitative data were collected through an open-ended question, and we could thus not explore themes deeper than what the participants chose to disclose through the survey. Additional qualitative research using more in-depth data is thus warranted. It is also important to note that the participants were likely more active than the general population, which may affect the overall generalizability of these findings. Lastly, although ESSES and 3 × 2 AGQ-S demonstrated acceptable internal consistency, the exploratory factor analyses did not fully replicate the original factor structures, which may indicate sample-specific or translation-related influences on the psychometric properties of the scales.

## 5. Conclusions

Strava may both support training and introduce motivational pressures in runners. In this sample of active club runners, self-efficacy and achievement goals were generally high, and estimates of aerobic endurance were associated with more adaptive motivational profiles. Strava was described by participants as fostering perceived competence, continuity, and social connectedness, while also exposing users to comparison, evaluation, and stress, particularly in periods of reduced performance or injury. The impact of Strava on motivational dynamics may not only be shaped by its features, but also by how runners regulate their engagement with the application and their stage of training or life. Coaches and practitioners may therefore consider encouraging athletes to use Strava primarily for self-referenced tracking and progress monitoring, while limiting social comparison when it becomes detrimental. During injury or reduced performance, temporary withdrawal or selective sharing may help preserve motivation for training. Overall, supporting athletes in developing intentional and flexible Strava use strategies may help them benefit from the platform while avoiding its potential motivational downsides.

## Figures and Tables

**Table 1:** Participant characteristics.

	Women		Men		
	Mean ± SD	N	Mean ± SD	N	*p*-Value
Age (y)	37.25 ± 8.75	68	41.86 ± 10.59	157	0.003
Active running experience (y)	8.82 ± 8.58	68	10.92 ± 8.07	154	0.306
_WE_$\dot{V}$O_2max_ (mL·kg^−1^·min^−1^)	51.60 ± 7.03	68	56.38 ± 7.05	155	<0.001
Running volume (hours·week^−1^)	5.05 ± 2.71	68	5.21 ± 2.46	156	0.488
Years on Strava (y)	3.87 ± 2.66	68	5.82 ± 3.47	157	<0.001
Strava use (hours·week^−1^)	1:10 ± 1:06	54	1:16 ± 1:17	144	0.851
Low-intensity training (%)	50 ± 21	68	53 ± 23	157	0.488
Moderate-intensity training (%)	31 ± 16	68	32 ± 19	157	0.915
High-intensity training (%)	19 ± 14	68	18 ± 15	157	0.287

SD, standard deviation; N, sample size; _WE_$\dot{V}$O_2max_, watch-estimated maximal oxygen uptake; groups were compared with the Mann–Whitney U test; intensity fractions are calculated from self-reported training intensity based on the 5-zone model, where low-intensity = zone 1 and 2, moderate intensity = zone 3, and high intensity = zone 4 and 5.

**Table 2:** Correlations between self-efficacy, achievement goals, Strava use, fitness, and age.

	α	1	2	3	4	5	6	7	8	9	10	11	12
1. ESSES	0.89												
2. Task-approach	0.74	0.22 **											
3. Task-avoidance	0.83	−0.07	0.42 **										
4. Self-approach	0.74	−0.04	0.49 **	0.54 **									
5. Self-avoidance	0.91	−0.18 **	0.36 **	0.67 **	0.76 **								
6. Other-approach	0.90	−0.05	0.43 **	0.39 **	0.40 **	0.38 **							
7. Other-avoidance	0.94	−0.18 **	0.31 **	0.51 **	0.40 **	0.50 **	0.87 **						
8. Years on Strava		0.17 **	−0.02	0.01	−0.12	−0.20 **	−0.05	−0.05					
9. Strava screentime		−0.06	0.11	−0.04	0.09	0.02	0.06	0.02	−0.054				
10. Strava followers		0.28 **	0.21 **	0.04	0.01	−0.14 *	0.06	−0.01	0.52 **	0.27 **			
11. Strava following		0.24 **	0.21 **	0.03	0.03	−0.13	0.05	−0.01	0.46 **	0.32 **	0.95 **		
12. _WE_$\dot{V}$O_2max_		0.39 **	0.35 **	0.04	0.08	−0.06	0.08	−0.05	0.24 **	0.17 *	0.36 **	0.35 **	
13. Age		0.02	−0.19 **	0.02	−0.27 **	−0.16 *	−0.09	−0.07	−0.19 **	−0.06	−0.07	−0.08	−0.21 **

Note: α, Cronbach’s alpha; ESSES, Endurance Sport Self-Efficacy Scale; _WE_$\dot{V}$O_2max_, watch-estimated maximal oxygen uptake; * *p* < 0.05, ** *p* < 0.01.

## Data Availability

The data presented in this study are available on request from the corresponding author.

## Abbreviations

The following abbreviations are used in this manuscript:

3 × 2 AGQ-S	3 × 2 Achievement Goal Questionnaire for Sport
α	Cronbach’s alpha
EFA	Exploratory factor analysis
ESSES	Endurance Sport Self-Efficacy Scale
N	Sample size
NIF	Norwegian Olympic and Paralympic Committee and Confederation of Sports
r_s_	Spearman’s rank correlation coefficient
SD	Standard deviation
$\dot{V}$O_2max_	Maximal oxygen uptake
_WE_$\dot{V}$O_2max_	Watch-estimated maximal oxygen uptake

## References

[B1-behavsci-16-00224] Ames C., Archer J. (1988). Achievement goals in the classroom: Students’ learning strategies and motivation processes. Journal of Educational Psychology.

[B2-behavsci-16-00224] Anstiss P. A., Meijen C., Madigan D. J., Marcora S. M. (2018). Development and initial validation of the endurance sport self-efficacy scale (ESSES). Psychology of Sport and Exercise.

[B3-behavsci-16-00224] Bandura A. (1977). Self-efficacy: Toward a unifying theory of behavioral change. Psychological Review.

[B4-behavsci-16-00224] Braun V., Clarke V. (2022). Thematic analysis: A practical guide.

[B5-behavsci-16-00224] Brevig E. A., Mehus I., Williams J. M., Øvretveit K. (2024). Motivational dynamics and training experiences among female Brazilian jiu-jitsu practitioners. Martial Arts Studies.

[B6-behavsci-16-00224] Carrier B., Marten Chaves S., Navalta J. W. (2025). Validation of aerobic capacity (VO_2_max) and pulse oximetry in wearable technology. Sensors.

[B7-behavsci-16-00224] Davidson T. (2024). You can now pay a “mule” to earn your kudos—We went inside the murky world of Strava jockeys. Cycling Weekly.

[B8-behavsci-16-00224] Delrue J., Mouratidis A., Haerens L., De Muynck G.-J., Aelterman N., Vansteenkiste M. (2016). Intrapersonal achievement goals and underlying reasons among long distance runners: Their relation with race experience, self-talk, and running time. Psychologica Belgica.

[B9-behavsci-16-00224] Dien J. (2010). Evaluating two-step PCA of ERP data with geomin, infomax, oblimin, promax, and varimax rotations. Psychophysiology.

[B10-behavsci-16-00224] Elliot A. J., McGregor H. A. (2001). A 2 × 2 achievement goal framework. Journal of Personality and Social Psychology.

[B11-behavsci-16-00224] Elliot A. J., Murayama K., Pekrun R. (2011). A 3 × 2 achievement goal model. Journal of Educational Psychology.

[B12-behavsci-16-00224] Engel F. A., Masur L., Sperlich B., Düking P. (2025). Validity of $\dot{V}$O_2_max estimates from the Forerunner 245 smartwatch in highly vs. moderately trained endurance athletes. European Journal of Applied Physiology.

[B13-behavsci-16-00224] Franken R., Bekhuis H., Tolsma J. (2023). Kudos make you run! How runners influence each other on the online social network Strava. Social Networks.

[B14-behavsci-16-00224] Grieder S., Steiner M. D. (2022). Algorithmic jingle jungle: A comparison of implementations of principal axis factoring and promax rotation in R and SPSS. Behavior Research Methods.

[B15-behavsci-16-00224] Hively K., El-Alayli A. (2014). “You throw like a girl”: The effect of stereotype threat on women’s athletic performance and gender stereotypes. Psychology of Sport and Exercise.

[B16-behavsci-16-00224] Horcajo J., Santos D., Higuero G. (2022). The effects of self-efficacy on physical and cognitive performance: An analysis of meta-certainty. Psychology of Sport and Exercise.

[B17-behavsci-16-00224] Hulteen R. M., Smith J. J., Morgan P. J., Barnett L. M., Hallal P. C., Colyvas K., Lubans D. R. (2017). Global participation in sport and leisure-time physical activities: A systematic review and meta-analysis. Journal of Science and Medicine in Sport.

[B18-behavsci-16-00224] Janssen M., Scheerder J., Thibaut E., Brombacher A., Vos S., Guilhem G. (2017). Who uses running apps and sports watches? Determinants and consumer profiles of event runners’ usage of running-related smartphone applications and sports watches. PLoS ONE.

[B19-behavsci-16-00224] Karahanoğlu A., Gouveia R., Reenalda J., Ludden G. (2021). How are sports-trackers used by runners? Running-related data, personal goals, and self-tracking in running. Sensors.

[B20-behavsci-16-00224] Knechtle B., Nikolaidis P. T., Onywera V. O., Zingg M. A., Rosemann T., Rüst C. A. (2016). Male and female Ethiopian and Kenyan runners are the fastest and the youngest in both half and full marathon. SpringerPlus.

[B21-behavsci-16-00224] Kuru A. (2016). Exploring experience of runners with sports tracking technology. International Journal of Human–Computer Interaction.

[B22-behavsci-16-00224] Lochbaum M., Zanatta T., Kazak Z. (2020). The 2 × 2 achievement goals in sport and physical activity contexts. European Journal of Investigation in Health, Psychology and Education.

[B23-behavsci-16-00224] Mascret N., Elliot A. J., Cury F. (2015). Extending the 3 × 2 achievement goal model to the sport domain: The 3 × 2 achievement goal questionnaire for sport. Psychology of Sport and Exercise.

[B24-behavsci-16-00224] Montes J., Wulf G., Navalta J. W. (2018). Maximal aerobic capacity can be increased by enhancing performers’ expectancies. Journal of Sports Medicine and Physical Fitness.

[B25-behavsci-16-00224] Øvretveit K. (2020). Capacity and confidence: What can be gleaned from the link between perceived and actual physical ability in Brazilian jiu-jitsu practitioners?. Martial Arts Studies.

[B26-behavsci-16-00224] Petersen J. M., Kemps E., Lewis L. K., Prichard I. (2020). Associations between commercial app use and physical activity: Cross-sectional study. Journal of Medical Internet Research.

[B27-behavsci-16-00224] Russell H. C., Potts C., Nelson E. (2023). “If it’s not on Strava it didn’t happen”: Perceived psychosocial implications of Strava use in collegiate club runners. Recreational Sports Journal.

[B28-behavsci-16-00224] Strava (2025). About us.

[B29-behavsci-16-00224] The Cooper Institute (2017). Physical fitness assessment and norms for adults.

[B30-behavsci-16-00224] Tulle E., Bowness J., McKendrick J. H. (2018). Strava-using parkrunners: A community study.

[B31-behavsci-16-00224] Whelan E., Clohessy T. (2021). How the social dimension of fitness apps can enhance and undermine wellbeing: A dual model of passion perspective. Information Technology & People.

[B32-behavsci-16-00224] Wiley E., Moncion K., Rodrigues L., Fang H., Noguchi K. S., Roig M., Richardson J., MacDermid J. C., Tang A. (2024). Exploring differences between gender expressions in exercise self-efficacy and outcome expectations for exercise in individuals with stroke. PLoS ONE.

[B33-behavsci-16-00224] Wilke J., Vogel O., Vogt L. (2019). Why are you running and does it hurt? Pain, motivations and beliefs about injury prevention among participants of a large-scale public running event. International Journal of Environmental Research and Public Health.

[B34-behavsci-16-00224] Xiang P., Bruene A., McBride R. E. (2004). Using achievement goal theory to assess an elementary physical education running program. Journal of School Health.

